# Functional inhibition related to structure of a highly potent insulin-specific CD8 T cell clone using altered peptide ligands

**DOI:** 10.1002/eji.200737762

**Published:** 2008-01

**Authors:** Liliana G Petrich de Marquesini, Antonis K Moustakas, Ian J Thomas, Li Wen, George K Papadopoulos, F Susan Wong

**Affiliations:** 1 Department of Cellular and Molecular Medicine, School of Medical Sciences, University of BristolBristol, UK; 2 Department of Organic Farming, Technological Educational Institute of Ionian IslandsArgostoli Cephallonia, Greece; 3 Department of Internal Medicine, Section of Endocrinology, Yale School of MedicineNew Haven, CT, USA; 4 Laboratory of Biochemistry and Biophysics, Faculty of Agricultural Technology, Epirus Institute of TechnologyArta, Greece

**Keywords:** Antigens/peptides/epitopes, Autoimmunity, Cytotoxic, T cell receptors

## Abstract

Insulin-reactive CD8 T cells are amongst the earliest islet-infiltrating CD8 T cells in NOD mice. Cloned insulin B15–23-reactive cells (designated G9C8), restricted by H-2K^d^, are highly diabetogenic. We used altered peptide ligands (APL) substituted at TCR contact sites, positions (p)6 and 8, to investigate G9C8 T cell function and correlated this with structure. Cytotoxicity and IFN-γ production assays revealed that p6G and p8R could not be replaced by any naturally occurring amino acid without abrogating recognition and functional response by the G9C8 clone. When tested for antagonist activity with APL differing from the native peptide at either of these positions, the peptide variants, G6H and R8L showed the capacity to reduce the agonist response to the native peptide. The antagonist activity in cytotoxicity and IFN-γ production assays can be correlated with conformational changes induced by different structures of the MHC-peptide complexes, shown by molecular modeling. We conclude that p6 and p8 of the insulin B15–23 peptide are very important for TCR stimulation of this clone and no substitutions are tolerated at these positions in the peptide. This is important in considering the therapeutic use of peptides as APL that encompass both CD4 and CD8 epitopes of insulin.

## Introduction

Altered peptide ligands (APL) are peptide variants of the agonist peptide that are substituted at TCR contact sites 1–3. More rarely, peptides with substitutions at MHC binding sites have also shown interesting properties as APL 4, 5 such as stimulation of partial agonist responses 5. However, even though APL are very close in amino acid sequence to the native peptide, they induce a differential T cell function profile 2, 6. Some APL can induce some of the T cell functions induced by the agonist peptide, but not others, *i.e*. cytotoxicity or production of cytokines without proliferation 1. Others can induce a different cytokine profile 7, induce anergy 1, 8, or inhibit the agonist response 9, 10. Partial agonists can induce some but not all the agonist T cell responses; antagonists inhibit the agonist response in presence of the native peptide; and null ligands are not recognized by the TCR 3.

Studies in both CD4 and CD8 T cell systems have shown that APL can behave as antagonists and affect several cell functions including cytotoxic responses, cytokine production, serine esterase release, phospho-inositol hydrolysis and calcium influx 10–12. Investigations using superantigen and peptide antigen that bind to different sites of the MHC molecules, indicated that in some instances, APL could antagonize a superantigen-mediated T cell response ruling out MHC competition in those examples of APL antagonism 13.

We have previously isolated a highly pathogenic CD8 T cell clone, G9C8, from the NOD mouse that recognizes the insulin (Ins) Β15–23 peptide 14, 15. The peptide is within the B9–23 region that is autoantigenic for CD4 T cells both in the NOD mouse and in man 16, 17 and an APL has been used in an immunotherapy trial 18. The B15–23 peptide sequence, LYLVCGERG is unusual because peptides presented by K^d^ often have a large hydrophobic residue such as leucine or isoleucine at position 9 (p9) 19 whereas this peptide has glycine, the smallest amino acid.

The motif for H-2K^d^ predicts that there should be an aromatic residue at p2, such as tyrosine or phenylalanine, and a large hydrophobic aliphatic residue at p9 19, 20. Structural homology modeling has shown that the native Ins B15–23 peptide fits into the groove with the p2Y providing the primary anchor, and the p9G the secondary anchor, mainly through its free carboxylate end 21. Only a few peptide residues are responsible for the specific recognition by the TCR 2. Those amino acids within the peptide sequence that are recognized by specific TCR and play a crucial role in the TCR/MHC-peptide binding are known as primary contact sites 2. Secondary contact sites are also involved in this interaction but play a lesser role in TCR recognition 2.

In this study, we have identified Ins B15–23 APL that antagonize the TCR of this highly pathogenic CD8 T cell clone, G9C8, focusing on APL for Ins B15–23 at p6 and p8 of the nonamer peptide, since these are important TCR contact sites 21. None of the peptides altered at these positions binds any better to the MHC, nor do they stimulate any response from the cloned T cells. Furthermore, many of them are able to antagonize cytotoxic responses and IFN-γ responses. We have focused on the substitutions G6H and R8L and examine structural reasons why this may be so and correlate the structure with the functional response.

## Results

### Stimulation assays

Our previous studies had shown that conformation of the region around residues p6 and p8 of the Ins B15–23 peptide is crucial, and not even conservative substitutions were tolerated by the cognate TCR 21. We therefore tested for cytotoxic function, IFN-γ production and proliferation when replacing the native peptide Ins B15–23 by every possible natural amino acid substitution at either p6 or p8.

**Figure 1:**
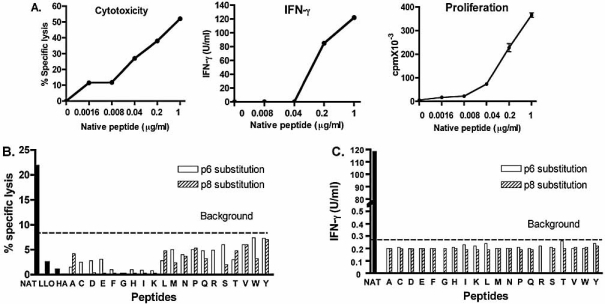
Cytotoxicity and IFN-γ production assays using native peptide and APL with substitutions at p6 and p8. (A) ^51^Cr-release cytotoxicity assay was performed using increasing concentrations of native Ins B15–23 peptide with P815 target cells and G9C8 cloned T cells as effectors at an E:T ratio of 10:1 following 4 h of incubation. IFN-γ production was measured by ELISA following incubation with P815 cells and increasing concentrations of B15–23 peptide after 24 h incubation. Proliferation is shown by [^3^H]thymidine incorporation in cpm following incubation of G9C8 cloned T cells, in triplicate, with increasing concentrations of Ins B15–23 peptide. (B) P815 target cells were first incubated with ^51^Cr-sodium chromate and secondly with different concentrations of native peptide (NAT), irrelevant control peptides (LLO and HA) and peptides altered at position 6 and position 8. The G9C8 cloned T cells as effectors were added to the plates at an E:T ratio of 10:1. Data are shown for a peptide concentration of 1 μg/mL. The background when effectors and targets were incubated in the absence of peptide was 8.6% in this assay. The data are presented as percentage of specific lysis. Each value corresponds to the average of duplicate samples. Results shown represent one of at least two independent assays. (C) G9C8 cloned T cells were stimulated by peptide-pulsed P815 cells at five peptide concentrations within the range 0.008–5 μg/mL. IFN-γ production at 1 μg/mL is shown. Supernatants were tested in duplicate taken after 24-h incubation at 37°C. Results correspond to one of two independent experiments. The limit of detection for the assay was 0.27 U/mL.

The G9C8 cloned T cells were used as effectors in a standard ^51^Cr-release assay against P815 target cells after incubation with six different concentrations of the peptides ranging from 0.0016–5 μg/mL (Fig. 1A). No stimulation was seen above the background (no peptide) for any of the substituted peptides or the two control peptides for any of the six concentrations of peptide tested (Fig. 1B and data not shown).

The G9C8 cells produce IFN-γ with increasing concentrations of peptide (Fig. 1A) and again, none of the p6 and p8 substituted peptides induced any cytokine production (Fig. 1C). Similarly, although G9C8 cells clearly proliferate to Ins B15–23 peptide (Fig. 1A), no proliferation was seen with any of the p6 or p8 substituted peptides (data not shown). This is in contrast to substitutions (even non-conservative ones) at p5, 7 and 9, where several substituted peptides were recognized in complex with H2-K^d^ at lower concentrations than the native peptide, by the G9C8 cells, resulting in cellular proliferation, IFN-γ secretion and cytotoxicity 21.

Thus, the systematic replacement of p6 and p8 of the native Ins B15–23 peptide by every other amino acid revealed that the TCR contact residues p6G and p8R could not be replaced without abrogating recognition by the G9C8 cells and loss of functional responses.

### Antagonist cytotoxicity and IFN-γ assays

**Figure 2:**
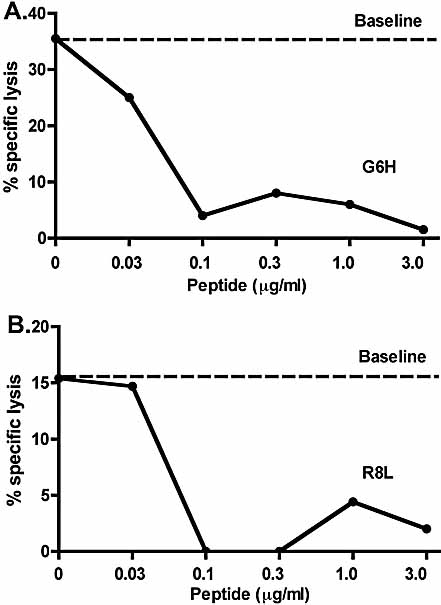
Antagonism of cytotoxic response with APL G6H (A) and R8L (B). P815 cells were incubated with ^51^Cr-sodium chromate and with Ins B15–23 (1 μg/mL). The cells were then washed, and further incubated with the APL G6H (A) and R8L (B) at concentrations ranging from 0.03 to 10 μg/mL. The G9C8 cloned T cells were then added to the plate at E:T ratios of 10:1 (A) and 8:1 (B). After 4-h incubation, supernatant was assayed for ^51^Cr release. Results were expressed as % specific lysis. The baseline shown here corresponds to the cytotoxicity to the APC preincubated with the native peptide but without addition of APL.

**Figure 3:**
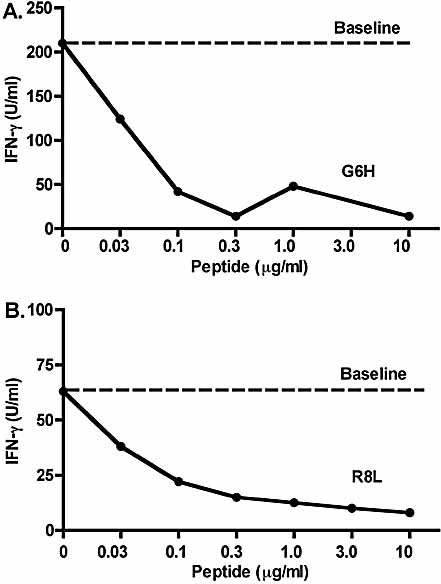
Antagonism of IFN-γ production using APL with substitution at p6 (A) and p8 (B). P815 cells were pulsed with 5 μg/mL of native peptide, washed, and then incubated with different concentrations of the peptide variants G6H (A) and R8L (B) (range 0.03–10 μg/mL). G9C8 cloned T cells were added for a further incubation of 24 h. IFN-γ release was determined by ELISA and results expressed in U/mL. The baseline shown here corresponds to the IFN-γ production of the APC preincubated with the native peptide but without addition of APL.

Each of the substituted peptides was tested in antagonist cytotoxic assays and IFN-γ production assays. All the peptide variants reduced lysis to a greater or lesser extent. However, three peptides (G6H, G6I, G6L) induce >50% antagonism at a peptide concentration of 0.1 μg/mL, a concentration >50 times lower than the native peptide concentration used in the assay. The antagonism shown by G6H is illustrated in Fig. 2A. The substituted p8 peptides were less effective, overall, although a similar antagonism effect in the assays was seen with R8L (Fig. 2B). Inhibition of IFN-γ production by the G6H and R8L mutant peptides is shown in Fig. 3.

### Binding assay results

**Figure 4:**
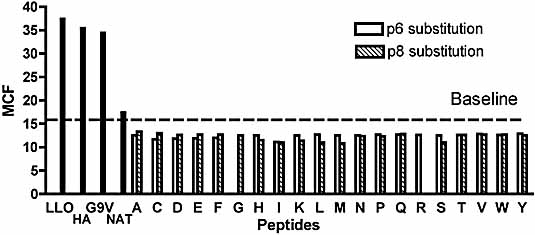
MHC-APL-binding assays of the APL with substitutions at p6 and p8 of the Ins B15–23 peptide. The binding of the APL at p6 and p8 to the MHC molecule is shown at a peptide concentration of 6.25 μg/mL. All the APL, for p6 or p8, fall below the baseline (given by the MHC binding of RMAS-K^d^ cells stained with the FITC-conjugated anti-K^d^ mAb but without the addition of any peptide), and the binding of the native peptide (NAT) is just above baseline levels. By contrast, good binding to the H-2K^d^ molecule is seen for the peptides LLO, HA and G9V (NAT with the residue valine substituting glycine at p9, previously shown to bind well to the MHC 21).

To evaluate the binding of the APL to the MHC, binding assays for each altered peptide at both p6 and p8 were performed and results are shown in Fig. 4. The binding of the APL at p6 and p8 of the Ins B15–23 peptide is extremely weak in this binding assay and no substituted peptide showed MHC-binding capacity greater than the native peptide. Thus, the ability of the peptides to antagonize the T cell functions is not related to competition for MHC binding with the native peptide as the altered peptides bound to the MHC more weakly compared to the native peptide.

## Molecular modeling

**Figure 5:**
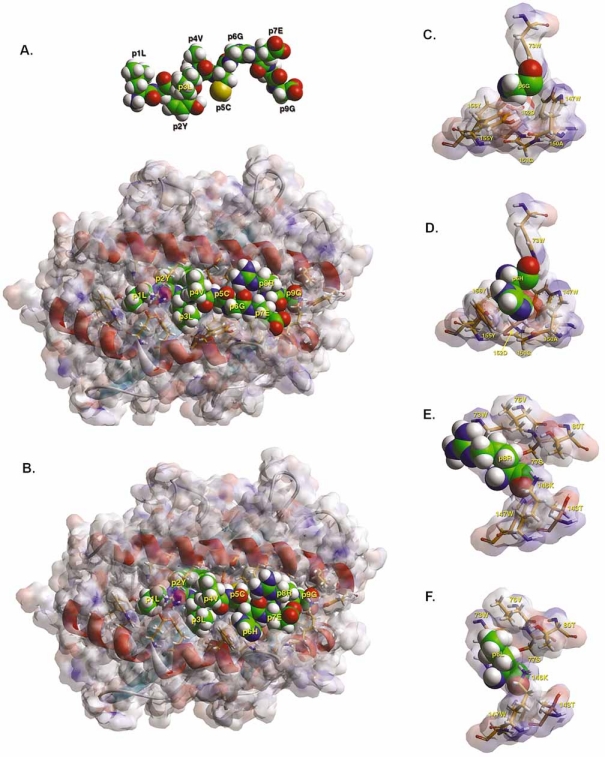
Molecular modeling of H-2K^d^−Ins B15–23 and mutated B15–23 peptides. (A) TCR view of the H-2K^d^−Ins B15–23 complex, and side view of the peptide chain as bound in the groove of H-2K^d^. This simulated structure is based on the crystal structure of H-2K^d^ in complex with an influenza nucleoprotein peptide 29. Several modes of structural rendering are shown simultaneously in order to appreciate how the peptide fits into the groove. The antigenic peptide is in space-filling form with its carbon atoms shown in green, nitrogen in blue, oxygen in red, hydrogen in white, and sulfur in orange. The α1α2 domain of the molecule is depicted according to its secondary structure in different regions: α-helix in red, β-pleated sheet in turquoise and random coil in grey. The solvent-accessible surface of the α1α2 domain is shown in grey with colorings according to the electrostatic surface potential (blue for positive, red for negative and intermediate hues for neutral). The surface of the heavy chain is made transparent so that peptide residue p2Y that is buried in pocket B, as well as the heavy chain residues making contact with the insulin peptide can be seen, albeit in a lighter color. These residues are shown with their carbon atoms in orange, while the color convention for the other atoms is identical to that for the antigenic peptide. (B) Modeled structure of the H-2K^d^ molecule with the Ins B15–23 p6H variant peptide, in the same orientation as the figure for the corresponding complex of the native peptide (A). Rendering and color conventions as in (A). (C) Molecular environment in p6 of the modeled complex with view of p6 containing the insulin peptide B15–23/B20G (p6G) residue and surrounding MHC heavy chain amino acids, in the complex of H-2K^d^ and Ins B15–23, as seen from above (TCR view). Color, surface electrostatic, and transparency conventions are as in (A). Some of the surrounding residues are at a distance longer than 4 Å, but are shown for comparison with (D). (D) View of p6 of the complex of H-2K^d^ with Ins B15–23/B20H (p6H), in the same orientation and conventions as in (C). All the surrounding residues from the heavy chain are at a distance of less than 4 Å. (E) View of p8 containing the Ins B15–23/B22R (p8R) residue and surrounding heavy chain amino acids, in the complex of H-2K^d^ and Ins B15–23, as seen from above (TCR view). Representations, color, surface electrostatic, and transparency conventions are as in (A). All the surrounding residues from the heavy chain are at a distance of less than 4 Å. (F) View of p8 of the complex of K^d^ with Ins B15–23/B22Leu (p8L), in the same orientation and conventions as in (E).

The results of molecular structural simulation, based on the crystal structure of a H2-K^d^ nucleoprotein peptide complex, show that the orientation of the Ins peptide in the groove is mostly the same (with the exception of residues p5C and p7E) as that obtained by modeling based on the HLA-A2-Tax peptide complex 21 (Fig. 5A). Remarkably, the crucial TCR contact residues p6G/p8R remain in nearly identical orientations as in the previous simulation (see below). There is a minor shift in the anchoring of the peptide, as it is now shown in this work, compared to previously 21, that the p5 residue in this peptide points into the groove (pocket C, 22). Furthermore, p7 is shown to be pointing towards the α2 helix and away from the groove, hence just outside the canonical “footprint” of the cognate T cell receptor 23. Assuming that the T cell receptor docks onto this complex in a diagonal manner as suggested by nearly all structures of such complexes, we conclude that p5C and p7E participate only slightly in TCR recognition 24–28. The position of p8R is nearly on the same level and in front of the α1 helix of H2-K^d^. We find it significant that in both molecular simulations, based on HLA-A2 21, and H2-K^d^ crystal structures (this work), the relative orientations of p6G and p8R are nearly identical. The residues p6G and p8R form a special pair such that any canonical TCR contact will necessarily involve both of them, as well as the peptide backbone of p7E (Fig. 5A).

It is apparent that even the slightest conservative alteration (*e.g.* p6G →A and/or p8R →K) will have important consequences on the canonical TCR affinity, as shown in the T cell recognition results. As seen in Fig. 5A, the Ins peptide backbone “leaps upward” from p6 to p8, relative to the β-sheet floor of the groove because residues 73W and 147W in the H2-K^d^ molecule, located across from each other, form a barrier over which the antigenic peptide must “climb” 29. Our molecular simulation has the guanidine side chain of p8R reaching backwards to p6G and being on the same level above the β-sheet floor. Such a unique arrangement would render these residues as prime contact residues for the canonical cognate TCR 25, 26, 28, 30. In the various substitutions at p6 and p8 modeled by molecular simulation, it has not been possible to duplicate this arrangement with any degree of physicochemical similarity. For example, the complex of H2-K^d^ with the p6H variant of the Ins peptide shows that p6H occupies prominent space previously left free for solvent and TCR contact, while the side chain of p8R is raised upward (Fig. 5B). Such significant changes would make it difficult for the cognate TCR to productively engage this variant complex in the same manner as the native peptide complex, as also verified experimentally (Fig. 1–3).

The comparisons of the native residues p6G and p8R with p6H and p8L are shown in Fig. 5C–F. Note that in the native complex, p8R is almost on the same level above the β-pleated sheet of the H2-K^d^ molecule as p6G, while p7E points to the side and p5C points into the groove (Fig. 5A).

Position 8 of the complex of K^d^ with Ins B15–23/B22Leu (p8L) in Fig. 5F shows the uniqueness of the native residue at this position. There is little change in the orientation of the proximal residues from H2-K^d^ and the side chain of p8L is essentially in the same orientation as the methylene groups of native p8R (Fig. 5E). The lack of the terminal guanidine group with its charged nitrogen makes all the difference as far as T cell recognition of this important residue is concerned.

## Discussion

The interaction of T cells with peptide-MHC complexes is of considerable importance both for positive and negative selection of mature T cells in the thymus as well as for activation of T cells in the periphery. In the thymus, antagonist peptides may induce or impair positive selection (reviewed in 31). Overall, the evidence suggests that positive selection is promoted by low-affinity self-peptides. Conversely, negative selection is triggered by high-avidity interactions, together with other influences outside the thymus 32. In the periphery, the mode of triggering of the TCR following binding to the MHC-peptide complex determines whether the cells are fully activated to perform effector functions, or partially stimulated or antagonized.

We have considered the properties of the Ins B15–23 peptide that stimulates a considerable percentage of CD8 T cells involved in the very early phases of pathogenesis of autoimmune diabetes in the NOD mouse 15. This peptide fits reasonably well into the H-2K^d^ groove with the p2Y providing the primary anchor, and the poor p9G the secondary anchor, mainly through its free carboxylate end 21, as expected for a peptide binding to the H-2K^d^ molecule. This would explain why the T cells require relatively high peptide concentrations to trigger effector functions. However, perhaps because of the relatively poor MHC binding of the peptide, thymic selection of the cells clearly occurs and these cells are found in the very early insulitis 33, even before other specificities, which become dominant, appear 34. When the cells encounter sufficiently high concentrations of peptide, as in the islets, they can show potent cytotoxicity (at concentrations of 40 ng/mL, Fig. 1). The concentrations of peptide that stimulate cytotoxicity are less than those required for IFN-γ production or for proliferation (200 ng/mL) as shown in Fig. 1.

Peptides that can bind to different MHC molecules use different residues to bind to the MHC or TCR as shown for the gp33 peptide of LCMV presented by both K^b^ and D^b^ molecules 35. Commensurate with this, APL used by LCMV to escape host defenses will have a different effect on the T cells that are restricted by different MHC types. For all antigenic peptides, such as the Ins B15–23 peptide, that bind to H2-K^d^, the conformation of the peptide in binding to the MHC molecule is important for determining the TCR contacts 2, 24–28, 36, 37. In the current study, we concentrated on the TCR contact residues 6 and 8, the two peptide residues not tolerant of even conservative substitutions, which point into the solvent and are high above other peptide residues, to look for antagonistic effects. In fact, most of the substitutions, to a greater or lesser extent antagonize the effector functions, but we have focused on those that are inhibitory at a low peptide concentration. Choice of antagonist peptide would be dictated by the lowest concentration of peptide that could inhibit both cytotoxicity and IFN-γ production. These peptides could potentially be used in the control of pathogenic G9C8-like CD8 T cells in damaging islet beta cells.

It is notable that the substitutions at p6 of the peptide have a greater antagonistic effect than those at p8, as more of the substitutions at p6 antagonize the cytotoxicity of the CD8 T cells. There are several possible reasons for this. P6G is a TCR-CDR3 contact point, and this can be the most crucial of peptide-TCR contacts. Furthermore, in this case, the p6 amino acid is glycine, the simplest, smallest and most flexible of amino acids. The peptide conformation in the MHC-peptide surface is likely to be very difficult to replicate with any amino acid substitution including alanine, which has the closest structure to the small glycine. It is highly likely that the same non-stimulatory or antagonistic effects would occur for any TCR that could recognize the K^d^-B15–23 MHC-peptide complex. In several studies, it has been shown that for particular MHC-peptide complexes, cognate TCR engagement crucially depends on the central peptide residue that is in contact with CDR3α and β of the TCR. In fact, especially for MHC class I-restricted recognition, alteration of a central residue might turn an agonist peptide into an antagonist 30. In the case of an autoimmune disease, there may be recognition of a putative initial pathogenic peptide-MHC combination by more than one type of TCR. In such cases, a single APL cannot be expected to “silence” all the pathogenic T cell clones as the exact orientation in the different TCR/peptide/MHC recognition complexes might differ 24. It might then be necessary to use a mixture of APL, each differing slightly from the native peptide, and using unnatural amino acids if necessary, in order to introduce the slightest of changes at putatively important TCR recognition sites 30. We also note that the concentrations of the antagonist peptide used in this case are 10–50 times lower than that of the native peptide, and as such compare well with the corresponding ratios in other MHC class I APL 38. Thus far there have been three autoimmune-related TCR-MHC II-peptide structures available, but none concerning MHC I complexes 39–41. The two human structures have the TCR in an off-diagonal mode of interaction, while the mouse structure is in the more common diagonal interaction. In all these, as well as in other cases, it is difficult to extrapolate from the mode and strength of TCR-MHC-peptide interaction the extent of reactivity/pathogenicity of the respective T cell clone 37.

The effect of peptide substitutions for a self-peptide involved in autoreactive responses leading to diabetes is important practically, as APL could potentially be used for human therapeutic trials in type 1 diabetes. Expression of altered insulin that includes a change in p2Y of the B15–23 insulin peptide has a profound effect on the recognition of autoreactive cells within the NOD mouse 42. It is most likely that this occurs because of alteration of the principal MHC-binding residue for the insulin-reactive CD8 T cell epitope as shown in our previous study 21, in addition to the effects that may occur for the CD4 T cells in that model system. The Ins B9–23 peptide that may bind to H2-A^g7^ of the NOD mouse MHC class II may do so in different registers, with B16Y either as p4 anchor, and identical in register to the HLA-DQ8-Ins complex 43, or as p5 TCR contact residue 44. Regardless of the exact core nonamer, it is very important to consider both CD4 and CD8 epitopes when proposing to use either native peptides or APL for immunotherapy. Furthermore, the detrimental effects of a cytotoxic epitope to an overlapping CD4 epitope for inducing tolerance in NOD mice have been shown for another region of the proinsulin molecule that encompasses both a CD4 (Ins B24–C32) and a CD8 (Ins B 25–C34) epitope 45. Recently, three independent studies have documented proinsulin-specific CD8 cells from peripheral blood of patients with type 1 diabetes who bear the most abundant human MHC class I protein (HLA-A2) 46–48. One of these peptides was the Ins B10–18 peptide that overlaps with the B13–21 core nonamer that binds to HLA-DQ8 and is recognized in that context by CD4 T cells from patients with type 1 diabetes 16. Whether proinsulin is the primary autoantigen in human type 1 diabetes remains to be established.

Our data suggest that any changes in p6 and p8 would have the added effect of actively reducing cytotoxicity and IFN-γ production of any Ins B15–23-reactive CD8 T cells. Unlike T cells recognizing other autoantigens such as islet-specific glucose-6-phosphatase catalytic subunit–related protein (IGRP), Ins B15–23-reactive CD8 T cells appear earlier in the pathogenic process and are detectable when insulitis first appears 15, 34. The dominance of proinsulin as the primary autoantigen in NOD mouse diabetes is shown by the fact that NOD mice made tolerant to IGRP are not protected from type 1 diabetes, compared with mice made tolerant to proinsulin 49. Unlike the studies on the prevalent IGRP-reactive cells that increase in number and avidity with time 50, these insulin-reactive T cells are of relatively low avidity and do not expand in the same manner. However, the insulin-reactive CD8 T cells are capable of being highly pathogenic 14 and the ability to antagonize such cells and prevent them from causing early islet damage is likely to add benefit in protection against diabetes. It may also be possible to administer APL in mannosylated form, thus enhancing their uptake by professional APC 100 to 1000-fold 51; these professional APC can also cross-present *via* the MHC class I pathway 52. In summary, our study provides both functional and structural evidence for the possible application of APL with mutated p6 and p8 of B15–23 in preventing diabetes. It is likely that this would have the greatest effect combined in a peptide that would also influence CD4 T cells. We will test this hypothesis *in vivo* in the near future.

## Materials and methods

### Clones and cell lines

#### T cell clones

G9C8 CD8 T cell clones were maintained in Bruff's medium (Invitrogen), supplemented with 5% FCS, and 5 U/mL IL-2 (supernatant from EL4 cell line), at 37°C in 5% CO_2_. The cells were fed every 2 weeks with islets isolated from young NOD mice by collagenase digestion, as previously described 14.

#### Cell lines

P815 cells are K^d^-expressing mast tumor cells. RMAS cells-K^d^ cells (kindly provided by A. Quinn of University of Toledo, USA) are a TAP-deficient lymphoma cell line, transfected with H2-K^d^. The MHC class I molecules are stabilized on the surface of these cells by bound peptide 53.

Both cell lines were maintained in complete medium (RPMI 1640 medium supplemented with 5% FCS, 2 mM L-Glutamine, 1% PSF and 50 μM 2-mercaptoethanol) at 37°C in 5% CO_2_.

#### Peptides

The native peptide Ins B15–23 (LYLVCGERG) was synthesized by the Keck facility, Yale University. The APL of the native peptide B15–23 substituted with all of the amino acids at p6 and p8, separately, were synthesized by Chiron Technologies, USA. The nomenclature adopted for the APL includes a letter, representing the native amino acid to be replaced; a number, representing the position in the peptide where the substitution takes place; and a second letter, representing the newly introduced amino acid.

### Assays

#### Chromium-release cytotoxicity assay

Chromium-release assays were performed as previously described 15. Target P815 cells were incubated with 0.1 μCi ^51^Cr-sodium chromate (Amersham) at 37°C for 1 h. The cells were then washed twice, and 10^4^ cells were further incubated with peptide concentrations ranging from 0.0016–5 μg/mL in round-bottom 96-well plates. The native peptide and each of the 19 peptides substituted at p6 and at p8 were tested together with two control peptides from listeriolysin (LLO) and hemagglutinin (HA). G9C8 cloned T cells (10^5^ cells) were added to the plates at an effector:target (E:T) ratio of 10:1. The assay mixture was incubated for 4 h at 37°C. Supernatant was assayed for ^51^Cr-release in a γ-counter. Results were expressed as % specific lysis determined as [(cytotoxic release - Min)/(Max - Min)] × 100, where the spontaneous lysis corresponds to the minimal release (Min), and the lysis provoked by addition of hydrochloric acid corresponds to the maximal lysis (Max).

#### Antagonist cytotoxicity assay

This is a modification of the standard cytotoxic assay described above, and based on two-stage antagonism assays 9. P815 target cells were incubated with ^51^Cr-sodium chromate and with a pre-titrated concentration (1–5 μg/mL) of the agonist peptide (Ins B15–23) at 37°C for 1 h. The cells were then washed twice and 10^4^ cells further incubated at 25°C with different APL at varying concentrations in 96-well plates for another hour. Then, 10^5^ G9C8 cloned T cells were added (effector-target ratio of 10:1). This E:T ratio was chosen following previous titration experiments. The assay mixture was incubated for 4 h at 37°C. Supernatant was assayed for ^51^Cr release in a γ-counter. Results were expressed as % specific lysis (% SL) as above. A further calculation was then performed to express % inhibition of lysis (% inhibition = ((Baseline (B) - cytotoxic release (X))/(Baseline - Min)) × 100.

#### T cell proliferation assay

NOD splenocytes (10^5^) as APC were irradiated (3000 rad) and incubated with native peptide (B15–23) at 25°C for 1 h. Cloned T cells (10^4^) were added and after 72 h incubation at 37°C, [^3^H]thymidine was added for a further 14–18-h incubation. Plates were harvested and [^3^H]thymidine uptake was measured in a β-counter.

#### IFN-γ assay

In IFN-γ production assays, P815 cells (10^5^) were incubated with native peptide (B15–23) at 25°C for 1 h. Cloned T cells (10^4^) were then added and after 24-h incubation at 37°C, the supernatant was harvested. IFN-γ production was measured by ELISA using antibodies purchased from BD Bioscience, using standard methodology 15. For the antagonist assays, P815 cells (10^5^) were incubated with native peptide at 25°C for 1 h. The cells were then washed, and incubated for another hour with different variant peptides at varying concentrations. Finally, cloned T cells (10^4^) were added and after 24-h incubation at 37°C, the supernatant was harvested and analyzed for IFN-γ production as above.

#### H-2K^d^-binding assay

H-2K^d^ is stabilized by peptide on the surface of RMAS-K^d^ cells. This is a standard assay for testing binding of MHC class I peptides to the MHC. The binding assay was performed as previously described 21. Briefly, RMAS-K^d^ cells were incubated at 30°C 54 for 16 h with four different concentrations of peptide (ranging from 0.39 to 25 μg/mL). Subsequently, cells were stained on ice with FITC-conjugated SF1–1.1 mAb (BD Bioscience) that stains H-2K^d^. Binding of peptide was measured by the degree of staining with SF1–1.1 and expressed as mean channel fluorescence (MCF) measured by flow cytometry.

### Molecular modeling

Structural homology modeling of the H-2K^d^ molecule with the Ins B15–23 peptide and its variants was performed, based on the crystal structure of the H-2K^d^-influenza A/PR8/34 nucleoprotein peptide 147–155 complex (29; PDB code 2FWO) as previously described, at an ambient pH value of 7.0 21, 55. Specifically, the Ins B15–23 residues (and APL where appropriate) were substituted by selecting the most suitable rotamer from a library of rotamers provided by the program Insight/Discover of Accelrys (San Diego, CA). Thereafter energy minimization of the H-2K^d^-Ins B15–23 complex was performed by the program Discover using 1000 steps of the steepest gradient method to be followed by 1000 steps of the conjugate gradient method. The decrease in energy was continuous with no abrupt changes. Extension of the conjugate gradient minimization to 10 000 steps (both here and in previous works 45, 55–57) yielded small decreases in the energy values with no significant shifts in the positions of MHC or antigenic peptide residues. No cross-terms were used in the potential function. Water molecules from the crystal structure were deleted and not taken into account for the minimization. This minimization procedure resulted in minimal change in the coordinates of Cα atoms of the H-2K^d^ molecule, in similarity to several previous works with MHC I and II molecules, with a root mean square deviation of K^d^ backbone atoms of ca. 0.51 Å for autologous (as here) or homologous molecules to 1.33 Å for xenogeneic MHC molecules (*e.g.* from HLA-A2 to H2-K^d^) 21, 45, 55–57. This approach is considered closer to the *in vivo* situation, where it has been shown that different peptides bound to the same MHC I molecule induce different conformations on the MHC residues contacting the peptide 22. Representations of all or part of the modeled H-2K^d^-Ins B15–23 complex were accomplished on a personal computer using the program DSViewer Pro Suite 6.0 of Accelrys. Unless otherwise stated, all representations of single antigenic residues, in particular positions, depict all heavy chain residues that have at least one atom at a distance of <4 Å from any atom of the particular peptide residue. The .pdb coordinates of the modeled H2-K^d^−Ins B15–23 complex and its variants are shown in Supporting Information.

## Abbreviations

APL: altered peptide ligand
IGRP: islet-specific glucose-6-phosphatase catalytic subunit–related protein
Ins: insulin
LLO: listeriolysin
p: position
